# Newer Hemostatic Agents Used in the Practice of Dermatologic Surgery

**DOI:** 10.1155/2013/279289

**Published:** 2013-08-07

**Authors:** Jill Henley, Jerry D. Brewer

**Affiliations:** ^1^College of Osteopathic Medicine Glendale, Midwestern University, 13989 N59th Avenue, Glendale, AZ 85308, USA; ^2^Division of Dermatologic Surgery, Department of Dermatology Mayo Clinic, Mayo Clinic College of Medicine Rochester, 200 First Street SW, Rochester, MN 55905, USA

## Abstract

Minor postoperative bleeding is the most common complication of cutaneous surgery. Because of the commonality of this complication, hemostasis is an important concept to address when considering dermatologic procedures. Patients that have a bleeding diathesis, an inherited/acquired coagulopathy, or who are on anticoagulant/antiplatelet medications pose a greater risk for bleeding complications during the postoperative period. Knowledge of these conditions preoperatively is of the utmost importance, allowing for proper preparation and prevention. Also, it is important to be aware of the various hemostatic modalities available, including electrocoagulation, which is among the most effective and widely used techniques. Prompt recognition of hematoma formation and knowledge of postoperative wound care can prevent further complications such as wound dehiscence, infection, or skin-graft necrosis, minimizing poor outcomes.

## 1. Introduction

Dermatologists are estimated to perform over 3.9 million procedures each year [1]. Although the risks and complications of dermatologic surgery are generally very low, even the most talented surgeon can experience complications related to hemostasis during both the intraoperative and postoperative periods. Minor bleeding complications are the most frequently encountered complications of cutaneous surgery, which can predispose the patient to hematoma formation, increased risk of infection, skin graft necrosis, and wound dehiscence. This chapter will highlight proper hemostasis technique to prevent complications.

## 2. Overview of Hemostasis

By understanding the mechanism behind the physiologic clotting system, it is easier to understand how the different hemostatic agents work in the body. The body's primary response to injury is reflex vasoconstriction of the blood vessels in the surrounding tissues, followed by formation of the platelet plug and activation of the fibrinolytic clotting cascade. There are two separate pathways of the fibrinolytic clotting system, that lead to the final common pathway and formation of the insoluble fibrin clot. Function of the body's hemostatic system can be monitored by various laboratory tests. These tests can be helpful to assess the degree of anticoagulation in patients on antiplatelet and anticoagulant medications or who have inherited coagulopathies before proceeding with dermatologic procedures (see Table 1).

## 3. Preoperative Evaluation

One of the most important steps that the dermatologist can take to prevent bleeding complications is to gain a thorough preoperative history of the patient before performing any kind of dermatologic procedure. This allows the physician to gain a better understanding of the patient's overall health and should include a detailed history of the patient's comorbidities, prior surgeries including complications, current medications, social history, and family history, which can help reveal any potential bleeding diatheses. 

 Specifically, questions regarding comorbid illnesses that can lead to coagulopathies, such as chronic renal and liver disease, hematologic disorders, and malignancies, should be asked, in addition to prior diagnoses of inherited bleeding disorders such as von Willebrand's disease and hemophilia [7]. Many mild forms of bleeding disorders go undiagnosed until later in life and should be investigated by thorough questioning of bleeding complications in prior minor surgical procedures (dental/oral surgery), prolonged episodes of epistaxis, menorrhagia, bruising, history of prior blood transfusions, and any family history of bleeding disorders. On physical exam, it is important to look for any indications of hemostatic abnormalities such as increased bruising or petechiae. For more details on how to manage a patient with a bleeding disorder, please see the section titled, “Approach to the Patient with a Bleeding Disorder.” It is also important to investigate the patient's cardiac history including the presence of a pacemaker or implantable cardiac defibrillator (estimated that 4% of Mohs patients are estimated to have one), because the use of certain electrosurgical agents used for hemostatic purposes may be prohibited in these patients [8]. Also, alcohol is a natural anticoagulant, and obtaining information regarding consumption could be beneficial as part of the preoperative evaluation. 

## 4. Pharmacologic Agents and Their Effects on Anticoagulation

Many patients that are going to have dermatologic surgery are on anticoagulant and antiplatelet medications. One question many healthcare providers face prior to cutaneous surgery is whether to continue anticoagulation medications prior to surgery. The discrepancy lies between keeping the patient on their current medication regimen, potentially increasing the patient's risk for bleeding complications during the perioperative period, or discontinuing their medication, which has now been proven to increase the patient's risk of life-threatening thromboembolic events during the postoperative period. For a list of some of the most widely used anticoagulant and antiplatelet medications today, including pharmacodynamics of the different medications, and various recommendations regarding usage during the perioperative period see Table 2.

Due to several clinical studies conducted in the past ten years, the general consensus between dermatologic surgeons has been to continue patients on their anticoagulant medications preoperatively, because the benefit of these medications significantly outweighs the risk of bleeding complications during or after procedure [1, 9, 12–18]. The overall risk of hemorrhagic complications in cutaneous surgeries, such as continuous bleeding or hematoma formation in a patient who is not on anticoagulant medications, is very low (1.4%) [18]. 

In a 2005 nationwide survey of Mohs surgeons, 66% were found to continue Warfarin during the perioperative period [19]. Some studies have shown that there is an increase in minor bleeding complications for patients taking Warfarin chronically, which includes: minor bleeding defined as bleeding less than 24 hours postoperatively, hematoma formation, bleeding that is controlled in the office setting, and bleeding controlled with manual compression [1, 20]. For patients taking Warfarin, checking the international normalized ratio (INR) within 48 hours to one week prior to the surgery can give the surgeon a better idea of the current magnitude of anticoagulation. It is generally recommended that the INR level be within the therapeutic range of 2–3.5 preoperatively [21]. There is a suggestion that the higher the INR level (especially >3.5), the higher the risk for hemorrhagic complications [15]. Because minor bleeding can be psychologically disturbing, an elevated INR level above the therapeutic range can be an indication for postponing the procedure depending on the urgency of the surgery. 

In comparison to Warfarin, the 2005 survey of Mohs surgeons found that a majority of surgeons (87%) discontinue prophylactic (not medically necessary) aspirin use 7–10 days prior to surgery, with a majority (77%) continuing medically necessary aspirin [19]. There is a consensus that the benefits of continuing medically necessary aspirin outweigh the risk of discontinuation. Patients taking another antiplatelet agent, Clopidogrel, have been found to be at increased risk of bleeding complications during cutaneous surgery. In a recent study conducted at Mayo Clinic, patients were found to be twenty eight times more likely to have a severe complication (defined as bleeding for <1 hour, bleeding not stopped with pressure, acute hematoma formation, flap or graft necrosis, or wound dehiscence >2 mm) with Clopidogrel use during surgery [13]. Patients were also found to be eight times more likely to experience a severe complication when taking Clopidogrel in combination with aspirin than aspirin monotherapy [13]. Although anatomical site has been speculated as a possible risk factor for postoperative bleeding complications, it appears that flaps and grafts are the biggest associated risk [13]. There have been reports in the literature of patients on anticoagulation medications who unfortunately developed either a thromboembolic stroke or acute myocardial infraction after stopping their anticoagulation medications to undergo cutaneous surgery [17]. Thus it is the current consensus of dermatologic surgeons in the United States, and the opinion of the authors, no matter what the increased risk of a postoperative bleeding complication might be whether influenced by anatomic site or anticoagulation, that these anticoagulation medications should not be stopped prior to cutaneous surgery regardless of the anatomic site or anticipated complicated nature of the cutaneous surgery. Although bleeding risk has been found to be significantly increased in these patients, continuation of the medication during surgery is still recommended due to the increased risk of life-threatening thromboembolic events that can accompany the discontinuation of these medications. 

Many patients are not just on one type of anticoagulation or antiplatelet agent but a combination. Certain combinations of anticoagulation medications, especially with Clopidogrel, have been shown to have a more profound effect on bleeding complications. Distinctly, the combination of Warfarin and Clopidogrel is 40 times more likely to lead to increased perioperative and postoperative bleeding complications, including hematoma formation in comparison to other anticoagulant agents [1]. Patients with recent drug eluding stent placement are advised to remain on dual antiplatelet therapy with Clopidogrel and aspirin for six months to one year and are highly advised to continue both of these medications in the perioperative period due to high risk of stent restenosis [22, 23].

Although most studies support continuation of anticoagulant medications perioperatively, a 2005 survey of 271 Mohs surgeons found that 37% still discontinued medically necessary aspirin and 44% still discontinued Warfarin [19]. Discontinuation of these medications can lead to life-threatening thromboembolic events such as deep venous thromboses, pulmonary embolism, myocardial infarctions, cerebrovascular accidents, cardiac stent thrombosis, or clotted prosthetic heart valves [17, 23]. Alam and Goldberg presented two cases that led to pulmonary embolus and a clotted prosthetic heart valve within 36 hours after operative cutaneous surgery due to the patients' antiplatelet and anticoagulant medications being discontinued [17]. It appears that prophylactic aspirin use can be discontinued 7–10 days prior to the procedure without significant risk of thrombotic events. 

## 5. Alternative Medicine and Herbal Supplements

According to the 2007 National Health Interview Survey, 4 out of 10 adults in the United States were found to have used some form of complementary alternative medicine during the past year. It has been shown that up to 70% of people do not tell their physicians that they are taking a herbal supplement [24]. Many popular alternative supplements contain a dietary ingredient, such as garlic, *ginkgo biloba*, feverfew, ginseng, and ginger [25]. Although it has become increasingly common for patients to be using alternative therapies such as those mentioned above, they are unlikely to volunteer this information to their physician [26]. Along with western pharmacotherapy, alternative therapies can have dose-related antiplatelet side effects especially in combination with other anticoagulant/antiplatelet pharmacologic agents (see Table 3).

## 6. Approach to the Patient with a Bleeding Disorder

Whether discovered upon taking a thorough preoperative history or a previously diagnosed condition, knowledge of an inherited or acquired bleeding disorder is of great importance before proceeding with dermatologic surgery. This section discusses how to approach patients with acquired disorders of coagulation due to chronic illnesses such as uremia secondary to chronic renal failure, severe liver cirrhosis, and the most commonly encountered hereditary bleeding disorders such as von Willebrand's disease and hemophilia A/B. 

The rates of chronic illnesses such as chronic renal failure are on the rise in the United States, with an increase in population longevity and chronic debilitating illnesses such as hypertension and type II diabetes mellitus. Uremia secondary to chronic renal failure causes a qualitative platelet defect that can lead to a bleeding diathesis and can be monitored by checking a bleeding time or PFA-100. Knowledge of this condition can help prevent bleeding complications, and by working in conjunction with the patient's nephrologist, the platelet defects can often be improved with hemodialysis or desmopressin prior to the procedure [31]. Desmopressin improves platelet defects, providing improvement of the bleeding time for up to 24 hours [32]. Severe liver cirrhosis can also cause a coagulopathy, leading to increased risk for bleeding complications. Liver damage decreases production of the clotting factors decreasing the body's ability to form a fibrin clot, and many patients will exhibit concurrent portal hypertension causing splenic sequestration of platelets and thrombocytopenia. The patient's risk for bleeding can be monitored preoperatively by the PT, aPTT, platelet count, and bleeding time. The patient's gastroenterologist should be consulted prior to the operation, and administration of recombinant tissue factor VIIa, fresh frozen plasma, or prothrombin complex concentrates may need to be given pre/postoperatively to help manage bleeding [33]. 

Although relatively rare in general, von Willebrand's disease is the most common inherited bleeding disorder affecting up to 1% of the population [34]. With the proper management of these conditions both pre- and postoperatively in collaboration with the patient's hematologist, the patient's risk for bleeding complications decreases substantially. The severity of the inherited defect (amount of clotting factor absent) corresponds to the amount of preoperative preparation needed. For minor surgeries, such as dermatologic surgery, it is generally recommended that coagulation factor levels approach 40–50% of normal serum levels before proceeding with the operation, with continued factor replacement 5–7 days postoperatively [34]. 

Although von Willebrand's disease comprises up to 1% of the population, significant bleeding has been shown to occur in only 10% of the affected patients [34]. Desmopressin is commonly given to patients with this disease preoperatively to help increase release of vWF from the endothelial cells [34]. Severely affected patients also exhibit decreased Factor VIII levels (20%) and can be given Factor VIII concentrates pre/postoperatively [34]. Hemophilia A is more common than hemophilia B, and this is due to a decrease or absence of Factor VIII (Hemophilia B has decreased Factor IX) and is most often discovered in childhood due to greater risk for developing deeper hemorrhages such as: hemarthroses, CNS bleeds, hematomas, or hematuria [31]. In conjunction with the patient's hematologist, factor VIII and IX concentrates can be given to the patient preoperatively and should be continued for up to 5–7 days postoperatively [34]. Hematoma formation is the most common complication for hemophiliacs, even with factor replacement, so patients should be monitored closely during the postoperative period (see Table 4).

The importance of the preoperative history and physical exam cannot be underestimated. If there is any suspicion by the surgeon that a bleeding diathesis is present, the patient should undergo further laboratory testing to assess coagulation status. 

## 7. Introduction to Hemostatic Agents

There are many different hemostatic modalities that can be implemented during surgical procedures. The specific types of modalities used depend upon the surgeon's preference, the efficacy and ease of use of the products, expense, and the bleeding risks of the particular patient at hand. The next few sections explore the various hemostatic techniques used today to provide optimal outcomes for the patient.

## 8. Anesthetic Techniques Promoting Hemostasis

The anesthetic agent chosen for the operation can provide hemostatic benefits to the patient when applicable. Anesthetic agents can lead to vasodilation of blood vessels increasing blood loss. Thus, the addition of a vasoconstrictive agent such as epinephrine or norepinephrine can improve intraoperative hemostasis. Not only do vasoconstrictive agents decrease bleeding, but they also increase the duration of action of the anesthetics, leading to decreased anesthetics required and prolonged anesthetic effect after procedure. Premixed concentrations of epinephrine in 1 : 100,000 or 1 : 200,000 are generally considered safe and effective [10]. Caution should be taken in pregnant and breastfeeding patients because epinephrine is considered Category C and can be secreted in breast milk [10]. Epinephrine infused anesthetic agents should also be used cautiously in patients who have vascular compromise or who take beta blockers. In patients on beta blockers, epinephrine leads to unopposed alpha_1_-receptor stimulation which can lead to life-threatening increases in blood pressure [11]. Epinephrine is absolutely contraindicated in patients with severe cardiovascular disease, severe hypertension, pheochromocytoma, or severe hyperthyroidism [10]. In the past, it was generally recommended that epinephrine be avoided in procedures involving the nose, ear lobes, fingers, toes, or genitals, including the penis, but recent studies have shown that certain concentrations of anesthetic with epinephrine (0.5% lidocaine with 1 : 200,000 epinephrine for example) show no evidence of ischemia or necrosis when injected into the digits [35]. Caution, however, should be used when operating on these special sites in patients with peripheral vascular disease and compromised circulation. 

Another anesthetic technique that provides excellent hemostasis is tumescent anesthesia. This technique is often used in liposuction surgery or in areas of the body associated with increased risk for bleeding. The technique is accomplished by injecting extremely dilute concentrations of lidocaine (0.1%) and epinephrine (1 : 1,000,000) subcutaneously into the tissues providing anesthesia to the superficial and deeper tissues while vasoconstricting the surrounding blood vessels [10, 36]. Dr. Jeffrey Klein, the innovator of the tumescent anesthesia technique, created the original formula, adding 1,000 mg of lidocaine and 1 amp of 1 : 1,000 epinephrine to one liter of normal saline, creating the concentration of 0.1% lidocaine with 1 : 1,000,000 epinephrine [10]. The large amount of fluid injected leads to swelling and induration of the tissues, placing pressure on the surrounding nerves and vascular structures, providing anesthetic and hemostatic effects on the tissue [36, 38]. For full hemostatic effect, the surgeon should wait 20–30 minutes after tumescent anesthesia is started before beginning with the procedure; however most of the time, the anesthetic effect of tumescence anesthesia is almost instantaneous, especially when it involves the superficial layers of the skin [10]. This technique provides prolonged anesthesia to the patient post-procedurally for up to 48 hours [37].

## 9. Electrosurgery

Electrosurgery is by far the most common hemostatic technique used in cutaneous surgery due to its accessibility, multi-functionality, ease of use, low expense, and effectiveness. There are different types of electrosurgical units depending on the surgeon's desired use of the product. The electrosurgical unit can be monoterminal or biterminal depending on the number of electrodes. The biterminal electrosurgical unit works by producing a high frequency, low voltage electrical current that is transmitted from an electrosurgical generator through an active electrode to the patient's tissues, and then back to the generator through a return electrode [10, 39]. The electrical current can be transmitted through the active electrode to the skin through one tissue contact point (monopolar) or through two tissue contact points (bipolar). An example of bipolar electrocoagulation would be the use of tissue forceps. Some studies suggest that the use of bipolar electrocoagulation produces less surrounding tissue damage, due to the ability of the forceps to grasp the specific hemorrhagic vessel providing hemostasis to a localized area [40]. The waveforms that are transmitted to the tissues can be categorized as damped or undamped. Damped waveforms provide the best hemostasis by generating heat to the tissues, leading to sealing of blood vessels; however these techniques can be more destructive [8]. 

The most common types of electrosurgery used by dermatologic surgeons are electrodesiccation, electrosection, electrocoagulation, and electrocautery [8]. A unit such as the Bovie, capable of conducting electrosection and electrocoagulation, provides the physician the ability to cut through the tissues and simultaneously provide hemostasis in a biterminal fashion. The unit produces high amplitude, low voltage currents that can be damped or undamped depending on the desire to cut or provide coagulation. Electrocoagulation produces damped waveforms providing excellent hemostasis, while electrosection produces mainly undamped waveforms that slice through tissue layers [8]. Because these methods generate an electrical current, they can alter implantable cardioverter defibrillators and pacemakers, potentially producing premature firing of the devices, generating arrhythmias, or causing asystole. A method called electrocautery can be used in this select group of patients, because it works by generating heat from a high resistance wire instead of producing electricity [8]. Less heat can be generated in areas of increased blood flow; therefore, electrocautery is generally only used for hemostasis of small cutaneous vessels. Pooling of blood in areas of increased blood flow not only hinders the visualization of the specific bleeding vessels to be cauterized but also decreases the effectiveness of the cauterization by decreasing the conduction of electrical current to the tissues. This problem can be prevented by dabbing the site with gauze or a cotton tipped applicator followed by quick cauterization. 

**Figure 1:**
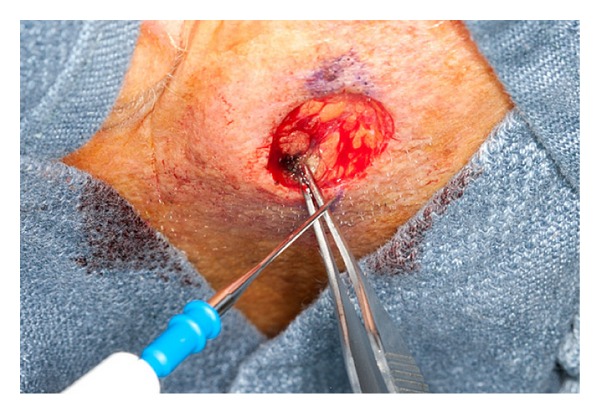
Minimizing the amount of surrounding tissue damage by using a monopolar electrocoagulation device applied to tissue forceps.

 Electrosurgery can produce thermal damage to the surrounding healthy tissues during the procedure [41]. Excess charring of the tissues can lead to decreased wound healing and slower recovery of the tissues postoperatively. This side-effect can be prevented by using the lowest power setting for the shortest amount of time during electrocoagulation or by touching a hemostat with the tip of a monopolar unit to produce pin-point coagulation (see Figure 1). Another potentially worrisome complication of electrosurgery is the risk of fire and electrical shock. This can be prevented by ensuring that the surgical area is not prepped with ethanol based products and through the use of insulated disposable tips on the electrosurgical device [10]. 35% aluminum chloride, a popular topical hemostatic agent used for shave biopsies, should not be used in conjunction with electrosurgery as well, because of the risk of fire. Also, the active electrode transmitting the electrical current to the tissue has an insulated shaft and base preventing electrical shock to the patient and surgeon [39]. It is important to keep in mind when removing lesions caused by human papillomavirus and other viral pathogens in the skin or when operating on patients who are suffering from a concomitant viral illness such as HIV or hepatitis, that these infections have the potential to be transmitted through the smoke plume [42]. Because of this, proper protection is warranted such as protective eye wear, masks, and gloves along with a smoke evacuator, and it is generally recommended that the smoke evacuator be held within 2 cm of the area being cauterized [42].

## 10. Physical Hemostatic Techniques

Among the cheapest and most accessible of all the hemostatic modalities is that of manual compression. This is by far one of the most basic techniques and has been used throughout history to stop bleeding and enhance coagulation. Downward pressure should be applied firmly to the affected area for 15–20 minutes depending on the extent of bleeding to tamponade the vessel(s). If the bleeding is severe, applying pressure to the supplying artery further upstream in addition to the wound area can help decrease blood flow to the affected area. This pressure applied allows time for platelets to adhere and initiation of the clotting cascade, as well as time to gather additional hemostatic agents to aid in the process. Sterile gauze pads and cotton tipped applicators can facilitate the process by soaking up the excess blood allowing better visualization of the surgical field and by providing counter pressure to aid in hemostasis. Cotton tipped applicators, (applicators with 8-inch handles and oversized cotton tips), are often used in nasal procedures, providing counterforce to the surgical field when entered in the nares for providing stabilization and hemostasis to the tissues [43]. Surgical instruments, chalazion clamp, can also provide hemostasis by manually compressing tissue during surgery [44, 45]. 

**Figure 2:**
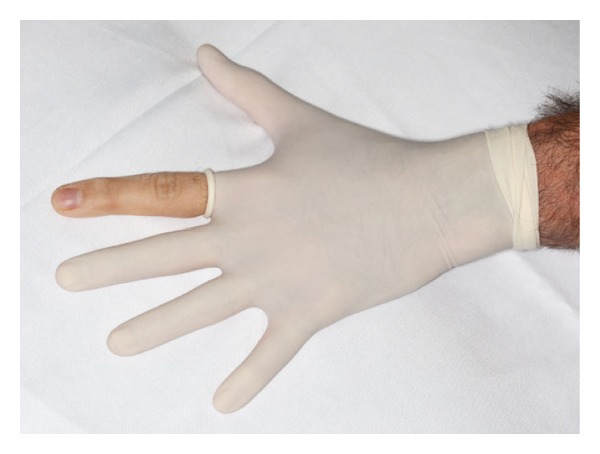
A surgical glove acting as a digital tourniquet.

Tourniquets have also been used to decrease blood flow to the procedural area. An example is a digital tourniquet made out of a single finger of a surgical glove. A small hole is pierced at the end of the finger of the glove, and the glove is rolled down the patient's digit, causing exsanguination. This digital tourniquet is then rolled tightly to the base of the metacarpophalangeal joint and stabilized with a hemostat that clamps and tightens the tourniquet [46] (see Figure 2).

**Figure 3:**
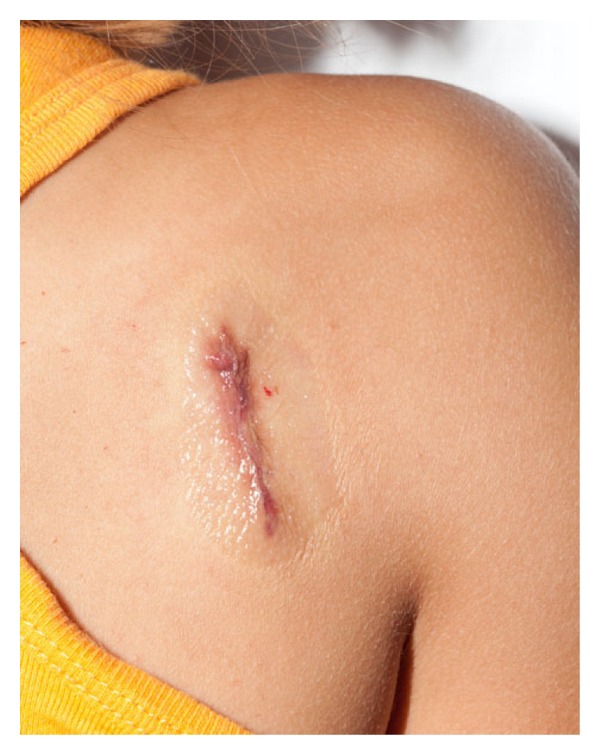
Dermabond liquid adhesive providing hemostasis to a child's laceration when applied topically.

Another physical method providing hemostasis for smaller wounds with minimal tension is through the use of acrylates. Octyl-2-cyanoacrylate (Dermabond) is a liquid that acts by polymerization to create a barrier, reaching its full strength in 2.5 minutes [47, 48]. This adhesive is approved for skin closure and is especially beneficial in children and patients with cognitive deficits who may not tolerate suturing or the removal of nonabsorbant sutures. Studies have shown other benefits with acrylates, including lower rates of bacterial contamination [47]. An animal study conducted in 2001 also showed earlier re-epithelization as well as decreased irritation with the use of Dermabond [49]. Another study showed that compared to using an adhesive bandage, acrylates provided significant hemostasis and pain relief. Band-Aid Liquid Bandage, an acrylate available over the counter, is a less expensive, more accessible option [48] (see Figure 3).

## 11. Suturing Techniques

 When performing punch biopsies and excisions of cutaneous lesions, preliminary sutures can be placed to decrease the hemorrhagic propensity of the procedure. One example is placing horizontal mattress sutures under the desired area prior to performing a punch biopsy. This can be very effective in areas that have an increased tendency to bleed such as the scalp [50]. 

 Another suturing technique that can be particularly useful for larger defects and removal of non-melanoma skin cancers is the purse-string technique [51]. This suture applies tension to the wound edge and compresses vessels in the reticular and papillary dermis decreasing bleeding complications [51]. 

 When bleeding from larger vessels (>2 mm) and cannot be controlled with manual compression or electrocoagulation, the vessels can be ligated or clamped with a hemostat. A figure-of-eight is most commonly placed around the vessel to tamponade the bleeding. 

## 12. Caustic Hemostatic Agents

This category of topical hemostatic agents is used with less frequency in dermatologic procedures today due to the corrosive effects that the agents have on the surrounding tissues. Caustic agents cause hemostasis by precipitating proteins in the tissues, causing occlusion of smaller vessels [48, 52]. One of the oldest topical hemostatic agents known as “Moh's paste” was created by Frederic Mohs in 1941 [53]. Zinc chloride is the main component of the paste, and it can be applied with a tongue depressor, cotton tipped applicator, or incorporated into gauze (product sold as Z-squares) [48]. Studies have shown that Moh's paste has been successful in providing hemostasis to friable tissues such as breast carcinomas that are metastatic to the skin [53]. 

 Another topical agent that is still used in dermatologic and gynecologic procedures, but with decreased frequency, is Monsel's solution, which is composed of 20% ferric subsulfate [48]. Monsel's solution has an acidic pH which is thought to contribute to its hemostatic properties eliciting protein precipitation in vessels and oxidization [51]. Monsel's solution is effective after punch or shave biopsies but is used less frequently due to its tattooing effect on the skin. Upon application, iron particles deposit into the dermis, leading to hyperpigmentation of the surrounding skin and an increased inflammatory response [48, 54]. The solution is user friendly and can be applied with gauze or cotton tipped applicators to the desired area and is relatively inexpensive. Less hyperpigmentation and tattooing of the skin occur with a 10% ferrous sulfate solution when applied to the skin for 1-2 hours postoperatively [48]. Another caustic agent that has a better side-effect profile than others is aluminum chloride. This solution can be applied with a cotton-tipped applicator after shave biopsies and has been shown to be very effective [48]. 

## 13. Noncaustic Hemostatic Agents

This group of hemostatic agents can be a helpful addition to electrocoagulation, minimizing the amount of electrocoagulation needed during the procedure. These are beneficial alternatives to patients with a bleeding diathesis despite use of electrocoagulation and decrease thermal injury to the surrounding tissues. This section is going to focus on various subsets of noncaustic hemostatic agents. A majority of these agents enhance the patient's own clotting system, so if the patient has a hereditary or acquired deficiency in the clotting cascade, these agents are unlikely to be beneficial (see Table 5).

Physical noncaustic agents work to provide a structural meshwork that aids in platelet aggregation and coagulation [48, 52]. An example of a physical agent is the gelatin sponge. Gelatin sponges have been used for hemostasis after punch biopsies of the skin and may be more effective in areas such as cartilage or periosteum, which have a harder time forming granulation tissue [48]. One study showed that wounds treated with gelatin sponges and left to heal by secondary intention led to increased granulation tissue formation and an overall better appearance of the wound [55]. Absorbable gelatin sponges soaked in aluminum chloride have also been shown to provide quick hemostasis after nail punch biopsies when left in the wound for two weeks [56]. Another physical agent, polyethylene glycol hydrogel, is a synthetic, biodegradable hemostat that polymerizes quickly, providing hemostasis in less than 60 seconds. Caution should be taken with closed wounds because it can swell up to four times its own size, potentially leading to surrounding tissue damage [57, 58]. Another physical agent, collagen, has been shown to be a superior hemostat to other products, because not only platelets adhere more readily to the collagen matrix, but also they are stimulated to degranulate enhancing platelet aggregation [57]. This product is less effective in patients with thrombocytopenia [58]. The product is bovine derived, with the potential to cause allergic and foreign body reactions. Other hemostatic agents containing oxidized cellulose (Surgicel or Oxycel), arranged into sheets or gauze, can be durably placed into bleeding tissues, causing hemostasis by tamponading vessels and by providing a physical meshwork for the clotting cascade to occur [48, 51]. Although relatively inexpensive and easy to use, they can potentially cause granulomatous reactions and increased swelling tissues [48, 60]. Two more physical agents, Urgent QR powder and microporous polysaccharide hemispheres, can be sprinkled topically on wounds to enhance hemostasis. Urgent QR powder is a hydrophilic polymer combined with potassium salt, used only on wounds left open to heal by secondary intention, because of the body's inability to metabolize the substance [52]. It hemostatically forms an eschar in the body in less than a minute, due to the polymers dehydrating the blood and the potassium salt binding to the positively charged red blood cells [48, 52]. It is sold over the counter and is less expensive than some of the other hemostatic agents [52]. Another physical agent, composed of purified potato starch powder, is microporous polysaccharide hemispheres. This agent can be degraded by enzymes in the body (alpha-amylase and pyrase), allowing use in closed wounds [52]. It accelerates the clotting process, by dehydrating the blood, causing concentration of the platelets and clotting factors [52, 58, 59]. This product can be sprinkled topically as a powder or incorporated into wound dressings. This agent has been shown to be less hemostatically effective and more expensive than electrocoagulation but is a good alternative in individuals that have contraindication to electrocoagulation [59]. This product should be used cautiously in diabetics, because it has the potential to increase glucose loads [58]. 

Physiologic agents are other hemostatic agents, which potentiate the body's own physiologic clotting mechanisms. For example, thrombin products have been created to enhance the fibrinolytic cascade and the final conversion of fibrinogen into fibrin. Topical bovine thrombin and human recombinant thrombin have been shown to be effective hemostatic agents in areas where there is diffuse bleeding (the specific vessel cannot be identified) and from direct bleeding from bone [61]. Studies have shown an increased risk for postoperative coagulopathies with bovine-derived thrombin, because antibodies formed against the thrombin are cross-reactive against human factor V [48, 57, 61]. Human recombinant thrombin has less antigenic effects, decreasing the risk for developing a postoperative coagulopathy, but carries a small risk for viral transmission to the patient [48, 57, 58, 61]. 

Another class of hemostatic agents gaining in popularity is the fibrin sealants. Whereas other products may rely on the patient's own platelet and clotting factors for hemostatic activation, fibrin sealants do not. This may be beneficial in situations where the patient has an inherited or acquired coagulation abnormality [62]. In order to be activated, the two compartments full of thrombin and fibrinogen must be mixed together (which can lead to clotting, if mixed prematurely) [48, 62]. When combined, thrombin (from one compartment) converts fibrinogen (from the other compartment) into insoluble fibrin in the presence of calcium [5, 48]. The amount of thrombin contained in the fibrin sealant is thought to contribute to the rapidity of clot formation, whereas the amount of fibrinogen contributes to the mechanical strength of the clot [58]. A 2009 Cochrane review showed that fibrin sealants lead to an average reduction of blood loss of 161 mL per procedure and argued that the benefits of using the product must outweigh the potential side effects of its use [5]. Potential side effects of fibrin sealants are infectious disease transmission, hypersensitivity reactions, and neurotoxicity [5, 48]. The newest fibrin sealant, Evicel, is human derived and does not contain tranexamic acid, decreasing the risk for neurotoxicity with use [58]. Newer fibrin sealant formulations are derived from autologous plasma or from pooled plasma donors, leading to decreased hypersensitivity reactions with the autologous sealants [5, 58]. 

 The authors recommend intraoperative hemostasis that is done with precision via electrosurgery as the mainstay of hemostasis in routine cutaneous surgeries. For smaller biopsies, where electrosurgery is not necessary, aluminum chloride is cheap and easily accessible and should also be considered as a mainstay. For postoperative bleeding complications, the other measures discussed in this section can be considered if physical hemostatic techniques (manual pressure) are inadequate.

## 14. Postoperative Recommendations and Complications

Patients that have increased intraoperative bleeding are at greater risk for postoperative bleeding complications, with complications most likely to occur within the first 48 hours following the procedure [10, 63]. A pressure dressing should be applied to the operative area for at least 24 hours postoperatively to ensure adequate compression of the tissues. The typical wound dressing normally consists of a topical antibiotic ointment, a Telfa pad cut to conform to the operative area, and a layer of gauze secured and compressed to the skin by adhesive tape [64]. It is generally recommended that patients avoid minor activity during the first 24 hours, allowing the vessels to remain coagulated, and to keep the wound site elevated for edema prevention. For procedures of the face, scalp, or neck, placing pillows underneath the head and neck areas while lying down can help to alleviate pressure and edema. Strenuous activity and heavy lifting should remain limited during the first month following the operation because the wound has only 40–50% of its tensile strength [64]. It is important to discuss with the patient that mild bleeding is anticipated, and if the patient starts to exhibit signs of increased bleeding, pressure and ice should be applied to the wound dressing for up to 20 minutes [63, 64]. Also, if the patient's wound dressing appears saturated with blood, it should be removed and a clean dressing should be applied to allow for better absorption and compression of the tissues. If the patient continues to have increased bleeding, the patient should return to the office for reevaluation. Alcohol ingestion is generally not recommended during the first week postoperatively due to ethanol's vasodilatory effects on the blood vessels leading to increased risk for bleeding (see Table 6).

Hematomas are most likely to occur within the first 24–72 hours postoperatively and can present with increased pressure sensation, throbbing pain, ecchymosis, and fluctuation of the tissues [10, 63]. Hematomas can form slowly due to continuous bleeding of smaller blood vessels into the newly closed wound or can expand rapidly when involving larger vessels. 

Early recognition of a rapidly expanding hematoma is important. Usually a hematoma is easily evaluated and diagnosed clinically as an expansile fluctuant mass under a recent surgical site that is accompanied by the characteristic expansile ecchymosis on the skin surface. If a hematoma is suspected clinically, the wound should be reopened and the affected vessels should be localized and treated with suture ligation or electrocoagulation. If bleeding continues despite prior attempts at coagulation, a drain can be placed in addition to other topical hemostatic agents [7, 10]. The drain should not be left for more than 24 hours, because of an increased risk for infection if left longer [65]. When hemostasis is achieved, all of the layers of the wound should be resutured closed and a clean pressure dressing should be reapplied. 

**Figure 4:**
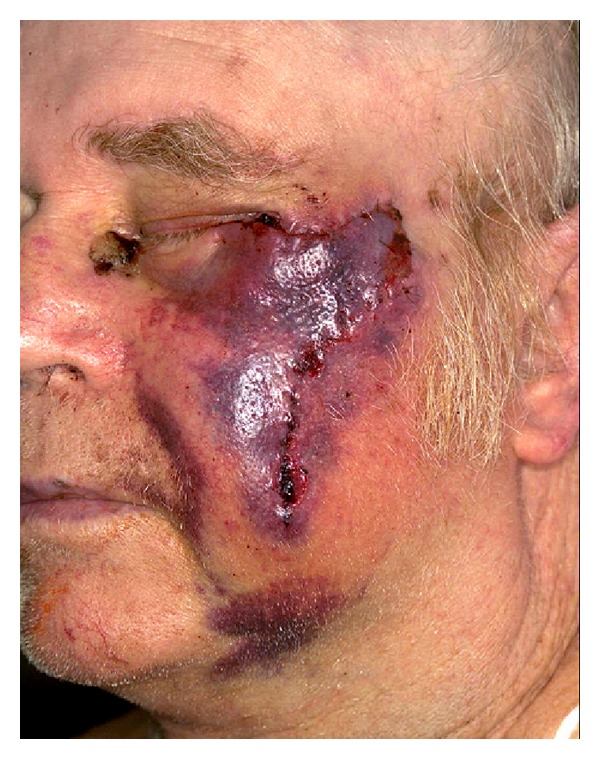
Acute hematoma formation following dermatologic surgery.

Hematomas become increasingly gelatinous and firm over time with the formation of clots. Patients who delay seeking treatment during the first week or who develop a hematoma very gradually can undergo observation or have their wound evacuated and left open to heal by secondary intention [63]. Weeks to months later, hematomas undergo liquefactive necrosis and resorption. During this stage, the hematoma can be aspirated and drained with a 16–18 gauge needle [63, 65]. Because hematomas are a nidus for bacteria, prophylactic antibiotics should be given early for infection prevention [7, 10]. If rapidly expanding hematomas are not controlled, they can lead to further complications such as wound dehiscence and skin graft necrosis (see Table 7 and Figure 4).

## 15. Summary

Hemostasis is an important concept to consider when performing dermatologic surgery. With careful attention paid to the preoperative evaluation, the patient's comorbidities and risk factors for bleeding, proper intraoperative hemostasis technique, and postprocedure monitoring, wound care, and education, many bleeding complications can be avoided or attended to promptly and effectively. During the procedure, many different hemostatic modalities are available, with electrocoagulation being among the most effective and commonly used. Many other methods of hemostasis can be used in conjunction with electrocoagulation with the optimal goal of minimizing blood loss. Postoperative complications due to increased bleeding include hematoma formation, skin or flap necrosis, and graft necrosis. Early evaluation of hematoma formation can help prevent the development of further complications such as infection, wound dehiscence, and skin graft necrosis. 

## Figures and Tables

**Table 1:** Overview of hemostasis.

Stages of hemostasis	Physiology	Monitoring
Primary hemostasis		
Formation of the platelet plug	Platelets first adhere to the exposed collagen and von Willebrand's factor on the subendothelium. Then, circulating stimuli activate the platelets, causing shape changes in the platelets [2]. Upon activation, platelet receptors get transferred to the surface, allowing for platelet aggregation. Platelets then release granules that stimulate further platelet aggregation and vasoconstriction [2, 3].	BT, PFA-100 analysis
Secondary hemostasis		
Intrinsic pathway	Plasma proteins get activated in contact with negatively charged surfaces, leading to activation of factor XII and other clotting factors, ultimately leading to the final common pathway and formation of the fibrin clot [4].	aPTT
Extrinsic pathway	Damaged endothelium exposes tissue factor, activating the extrinsic pathway, leading to thrombin production, and activation of other clotting factors, ultimately leading to the final common pathway and formation of the fibrin clot [2, 4].	PT
Final common pathway	Both pathways lead to activation of factor X, which converts prothrombin into thrombin. Thrombin leads to formation of the insoluble fibrin clot, by converting fibrinogen into fibrin [5]. The clot is then stabilized by factor XIII [6].	

Abbreviations: ADP: adenosine diphosphate, aPTT: activated partial thromboplastin time, BT: bleeding time, PFA-100 analysis: platelet function analysis, PT: prothrombin time, and TX_A_2__: thromboxane A_2_.

**Table 2:** Anticoagulant and antiplatelet medications [9–11].

Drug	Pharmacodynamics	Indications and monitoring	Discontinuation	Reversal
Warfarin	Coumarin inhibits the enzyme epoxide reductase, inhibiting the *γ*-carboxylation of Vitamin K-dependent clotting factors: II, VII, IX, X, Protein C, and S [11].	Indications: acute/chronic venous thromboembolism, pulmonary embolism, atrial fibrillation, prosthetic heart valves. Monitoring: PT and INR. When combined with aspirin, heparin, herbal supplements, or other acquired coagulopathies, can lead to potentiated increase in PT/INR in the patient. Generally recommend a PT/INR level 1 week prior to surgery (2–3.5 range recommended).	Discontinuation not recommended for dermatologic surgery. If patient is at high risk for bleeding during the procedure, consider delaying the surgery until better hemostatic control is obtained.	Reversal generally not needed. If an emergent situation arises, fresh frozen plasma, prothrombin complex concentrates, or recombinant Factor VIIa can be used. Parenteral Vitamin K administration can also be used, but takes longer for effects to be seen.

Unfractionated Heparin	Binds to antithrombin III which leads to inactivation of thrombin and Factor Xa.	Indications: DVT, pulmonary embolism, acute arterial occlusion, as a bridge in conjunction with Warfarin until Warfarin levels become therapeutic. Monitoring: aPTT. Check aPTT level 1 week prior to surgery as well as a CBC to check for platelet levels.	Discontinuation not recommended for dermatologic surgery.	Reversal generally not needed. If an emergent situation, 1 mg of protamine sulfate for every 100 units of heparin *in vivo* can be given.

Dabigatran	Direct thrombin inhibitor.	Indications: Atrial fibrillation. Monitoring: Not recommended.	Discontinuation not recommended for dermatologic surgery.	No reversal agent. Control bleeding site, give supportive care.

Aspirin	Irreversibly inhibits the cyclooxygenase enzymes, which decreases the levels of TX_A_2__, decreasing platelet aggregation.	Indications: MI, TIA/stroke prevention, CAD, fever, pain, inflammatory diseases (RA), cardiac stent placement. Monitoring: Not recommended, but platelet inhibition can be monitored by bleeding time or PFA-100.	Prophylactic aspirin with no prior history of myocardial infarction or cerebral vascular events should be discontinued 10–14 days prior to procedure due to irreversible effect on platelets and started 1 week postoperatively. ASA used for therapeutic purposes or prophylactically in high risk individuals should be continued.	Reversal generally not needed.

Ticlopidine and Clopidogrel	Thienopyridines that irreversibly inhibit adenosine-diphosphate receptors, decreasing platelet aggregation [12].	Indications: Drug eluding stent, TIA/stroke, MI, PVD. Monitoring: Not recommended. Can be monitored by bleeding time or PFA-100.	Discontinuation not recommended, although Clopidogrel has been shown to lead to greater bleeding complications than other antiplatelet agents [12].	Reversal generally not needed.

Cilostazol	Vasodilator that inhibits cellular phosphodiesterase, decreasing platelet aggregation.	Indications: Commonly used in treatment of peripheral arterial disease for intermittent claudication. Monitoring: Not recommended.	No recommendations have been made regarding discontinuation.	Reversal generally not needed.

Dipyridamole	Vasodilator that inhibits cGMP phosphodiesterase and cellular uptake of adenosine.	Indications: Mostly used in combination with other drugs such as aspirin (Aggrenox) or warfarin after cardiac valve replacement. Monitoring: Not recommended.	No recommendations have been made regarding discontinuation.	Reversal generally not needed.

NSAIDs (Ibuprofen, diclofenac)	Reversibly inhibit cyclooxygenase, inhibiting TX_A_2__, decreasing platelet aggregation.	Indications: Pain, inflammatory conditions (RA), fever, dysmenorrhea, HA. Monitoring: Not recommended.	Recommended to be discontinued 3–5 days preoperatively, with resumption 1 week post-operatively.	Reversal generally not needed.

Abbreviations: aPTT: activated partial thromboplastin time, ASA: aspirin, CAD: coronary artery disease, DVT: deep venous thrombosis, INR: international normalized ratio, MI: myocardial infarction, PVD: peripheral vascular disease, PFA-100: platelet function analyzer, PT: prothrombin time, RA: rheumatoid arthritis, TIA: transient ischemic attack, and TX_A_2__: Thromboxane A_2_.

**Table 3:** Dietary supplements and anticoagulant properties.

Type of supplement	Mechanism of action	Comments
Garlic	Allicin, adenosine, and paraffinic sulfide in garlic inhibit platelet aggregation, increasing bleeding time [26, 27].	Should be used in caution in conjunction with other anticoagulants such as Coumadin and heparin [27].
Ginkgo-biloba	Inhibits platelet activating factor [26]. Platelet aggregation thought to be inhibited by terpene ginkgolide B [24, 28].	Discontinue 36 hours before surgery [27]. One energy drink contains more than recommended dosage [28]. Caution should be used when combining with Cilostazil [28]. Some studies have shown no increase in bleeding when compared to a placebo [29].
Ginseng	Inhibits platelet aggregation by altering inhibiting thromboxane function [24, 27].	Large ingredient in energy drinks.
Ginger	Gingerol in ginger inhibits platelet function by inhibiting platelet activation also decreases synthesis of thromboxane [24, 27].	Has not shown to interact with NSAIDs or warfarin. More studies need to be performed on the extent of ginger's anticoagulant properties.
Vitamin E	Decreased platelet adhesion and aggregation [24].	Anticoagulant properties are dosedependent. Because it is a fat soluble vitamin, large doses can be stored in the body causing toxicity as well as increased propensity to bleed [27].
Omega-3-fish oil	Decreased platelet adhesion and aggregation [24].	Has not been shown to increase bleeding complications in spinal surgery [30]. In conjunction with other anticoagulant medications, may lead to increased effect [27].

**Table 4:** Acquired and inherited coagulopathies and management.

	Mechanism	Monitoring	Treatment
Acquired coagulopathies			
Uremia (chronic renal failure)	Qualitative defect in platelets with a normal platelet count.	BT or PFA-100	DDAVP; per patients nephrologist, hemodialysis, or peritoneal dialysis [31, 32].
Liver cirrhosis	Decreased production of the clotting factors; coincident splenomegaly can lead to sequestration of platelets and thrombocytopenia.	PT, aPTT, BT, and platelet count	Vitamin K, FFP, recombinant Factor VIIa, Cryoprecipitate, Platelet transfusions, Prothrombin complex concentrates, and Desmopressin [33].
Inherited coagulopathies			
Von-Willebrand's disease	Decreased production of von-Willebrand's factor and factor VIII.	BT, aPTT	DDAVP, factor VIII concentrates, Cryoprecipitate [34].
Hemophilia A	Decreased Factor VIII.	aPTT	Factor VIII concentrates, DDAVP [34].
Hemophilia B	Decreased factor IX.	aPTT	Factor IX concentrates [34].

Abbreviations: aPTT: activated partial thromboplastin time, BT: bleeding time, DDAVP: desmopressin, FFP: fresh frozen plasma, PFA-100: platelet function analyzer, and PT: prothrombin time.

**Table 5:** Hemostatic agents.

Hemostatic agent	Product information	Mechanism of action	Potential side effects
*Caustic agents *			
Zinc chloride (Moh's paste)	Paste that can be applied topically. Used infrequently, but is effective in providing hemostasis to metastatic cutaneous wounds [53].	Precipitates proteins causing coagulation of small vessels [48]. Left on for up to 48 hours.	Can be very painful and irritating to the patient [53].
Ferric subsulfate (Monsel's solution)	Solution can be applied with a cotton tipped applicator or gauze pad [48, 54].	Precipitates proteins intravascularly and oxidizes tissues. Less expensive, more accessible, and easy to apply [48, 54].	Being used less due to intradermal ferruginous deposits causing tattooing of the skin after use [48, 54].
Aluminum chloride	Solution can be applied with a cotton tipped applicator after shave biopsy [48].	Precipitates proteins causing coagulation of vessels [48]. Easily accessible and easy to apply.	Can be painful and irritating to the patient [48].
*Non-caustic agents *			
Gelatin (Gelfoam, Surgifoam) Gelfoam Plus (Gelatin combined with human thrombin)	Comes in a sterile powder or sponge. Porcine derived [48].	The gelatin is able to absorb more than 45x its weight, providing a matrix for the clotting cascade in addition to providing a physical barrier. Absorbed by the body in 4–6 weeks [48, 55, 56].	Can interfere with healing of wound edges, generally not recommended for use in skin incisions. Can facilitate bacterial growth leading to infection or leading to foreign body reactions when left in the tissue. Can increase in size leading to compression of surrounding structures, including nerve damage. When combined with thrombin can lead to allergic/anaphylactic reactions [48, 55].
Polyethylene glycol Hydrogel (CoSeal)	Liquid composed of two PEG polymers that polymerize and cross-link in the tissue [57, 58].	Increases platelet adherence, providing quick hemostasis [57].	Swells up to 4x its size potentially causing damage to the surrounding tissues [58].
Microporous polysaccharide spheres (Arista)	Comes in a white powder that is 100% plant based. Formed by cross-linking of purified plant starch [52, 58, 59].	Dehydrates the blood, concentrating RBC's, platelets, proteins, promoting adherence to the gel matrix. Also causes a physical barrier in the tissue [52, 58, 59].	Causes immediate swelling, has the potential to cause damage to surrounding structures. Use cautiously in diabetics due to potential to increase glucose load [58].
Microfibrillar collagen (Avitene, Helistat)	Bovine collagen formed into flour, sheets, or sponges [48].	Collagen framework promotes platelet aggregation and coagulation cascade [48].	Side effects are rare. Allergic and foreign body reactions have occurred [48].
Cellulose (Surgicel, Oxycel)	Oxidized cellulose arranged into sheets, gauze, or smaller strips. Can durably be placed in the tissue [48, 60].	Physically acts to tamponade the vessels and provide a meshwork for the fibrinolytic cascade to occur. Becomes gelatinous in 24–48 hrs and is absorbed by the body by 1–6 weeks. Relatively inexpensive [48].	Can cause granulomatous reactions and should be used carefully in closed spaces due to increased risk of swelling in the tissues, can cause compression of surrounding structures [60].
Pro QR powder	Combination of a hydrophilic polymer and potassium salt packaged into a powder [48, 51]. Available over the counter, relatively inexpensive and easy to apply.	Forms an eschar in body in <60 seconds, due to the polymers dehydrating the blood and the potassium salt binding to the positively charged red blood cells [48, 52].	Few side effects reported.
Thrombin (Thrombin-JMI, Recothromb, Evithrom, Floseal)	Can be bovine derived or human recombinant thrombin. Comes in a powder or solution. Floseal is composed of a gelatin matrix and thrombin and comes in two separate compartments that are not mixed until time of use [48, 60].	Promotes body's physiologic clotting cascade by actively converting fibrinogen into fibrin. Should not be used in patients that have decreased fibrinogen levels [48, 57, 60].	Bovine thrombin has been shown to cause coagulopathy weeks after use, due to antibodies forming against factor V. Human thrombin although cleansed thoroughly, has the potential to transmit viruses [48, 57, 60].
Fibrin sealant (Tisseal, Crosseal, Evicel)	Human and bovine derived forms. Can be formed from autologous plasma or pooled from donors. Also comes in an aerosolized form [5]. Entails different methods of preparation depending on the product. Can be frozen in premixed form for up to two years, can be heated, and stirred for 20 minutes prior to use [48, 60]. Can also be sprayed directly to the area in the aerosolized form [5].	Comes in a two compartment syringe with one compartment containing fibrinogen, factor XIII, fibronectin, and fibrinolysis inhibitors (aprotinin), and in the other is thrombin and calcium chloride [5, 48, 60]. When combined, thrombin becomes activated and activates the clotting cascade and enhances conversion of fibrinogen to fibrin. Absorbed from the body within 5–10 days [48]. Very effective in patients with coagulopathies.	Pooled donor plasma and older sealants containing bovine derived aprotinin increased the potential to cause hypersensitivity reactions and to transmit infectious diseases such as prion-related diseases [5, 58].
Octyl-2-cyanoacrylate (Dermabond)	Comes as a topical liquid adhesive best used for smaller lacerations. May have some antibacterial properties. Good in pediatric population and people with cognitive deficits who cannot tolerate/understand stitch removal. Moderately expensive [47–49].	Polymerization creates a physical barrier, tamponading the vessels [47–49].	Small risk for inflammatory reactions and fibrosis [48].

Abbreviations: PEG: polyethylene glycol.

**Table 6:** Postoperative recommendations.

Postoperative recommendations	
(i) A pressure wound dressing should be applied for at least 24 hours, providing adequate compression to the tissues [10, 63]. (ii) Activity should be limited during the first 48 hours due to the increased risk for bleeding complications. Small blood vessels are vulnerable to rupture with minor activity. Strenuous activity should be limited until the wound regains tensile strength (up to 2 weeks for facial/neck wounds and up to 6 weeks for lower extremity wounds) [10]. (iii) Elevation of the operative area within the first 24 hours of the operation is important to decrease the amount of gravity and pressure on the tissues. For procedures of the face, scalp, or neck, placing pillows underneath the head and neck areas while lying down can help to alleviate pressure [64].	(i) If bleeding is apparent, ice and cool compresses can be applied to the surgical area to vasoconstrict the blood vessels and decrease bleeding [64]. (ii) Manual compression can be applied externally to the wound area for up to 20 minutes to control minor bleeding. (iii) If the gauze and current wound dressing appear to be saturated in blood, a clean dressing should be applied to the area to allow for better absorption and compression of the tissues. (iv) If the patient begins to experience throbbing pain, increased pressure sensation, or heavy bleeding that is uncontrollable, they should return to the office immediately for further intervention and treatment [63].

**Table 7:** Hematoma management.

Early hematoma formation	Late hematoma formation
(i) Reopen the wound and localize the bleeding site. (ii) Achieve hemostasis by electrocoagulation/ligation of the affected vessels or by application of topical hemostatic agents. (iii) If bleeding cannot be adequately controlled, place a drain into the wound for up to 24 hours. (iv) Resuture the site and apply the appropriate pressure dressing.	(i) If small, may only require observation. (ii) If large and within the first week, evacuate the area and leave open to heal by secondary intention [63]. (iii) If not discovered until late (weeks to months later), aspirate the area with a 16–18 gauge needle; [63, 65].
